# Amphiregulin blockade decreases the levodopa‐induced dyskinesia in a 6‐hydroxydopamine Parkinson's disease mouse model

**DOI:** 10.1111/cns.14229

**Published:** 2023-04-26

**Authors:** Piniel Alphayo Kambey, Wen Ya Liu, Jiao Wu, Chuanxi Tang, Wokuheleza Buberwa, Adonira Saro, Alphonce M. K. Nyalali, Dianshuai Gao

**Affiliations:** ^1^ Xuzhou Key Laboratory of Neurobiology, Department of Neurobiology and Anatomy Xuzhou Medical University Xuzhou China; ^2^ Organization of African Academic Doctors (OAAD) Nairobi Kenya; ^3^ Department of Pediatrics The Second Affiliated Hospital of Xi'an Jiaotong University Xi'an China; ^4^ Department of Anatomy and Neurobiology, School of Basic Medical Science Central South University Changsha China; ^5^ Department of Neurosurgery, Shandong Cancer Hospital and Institute Shandong First Medical University and Shandong Academy of Medical Sciences Jinan China

**Keywords:** Amphiregulin, dyskinesia, levodopa, Parkinson's disease

## Abstract

**Background:**

Levodopa (L‐DOPA) is considered the most reliable drug for treating Parkinson's disease (PD) clinical symptoms. Regrettably, long‐term L‐DOPA therapy results in the emergence of drug‐induced abnormal involuntary movements (AIMs) in most PD patients. The mechanisms underlying motor fluctuations and dyskinesia induced by L‐DOPA (LID) are still perplexing.

**Methods:**

Here, we first performed the analysis on the microarray data set (GSE55096) from the gene expression omnibus (GEO) repository and identified the differentially expressed genes (DEGs) using linear models for microarray analysis (Limma) R packages from the Bioconductor project. 12 genes (*Nr4a2, Areg, Tinf2, Ptgs2, Pdlim1, Tes, Irf6, Tgfb1, Serpinb2, Lipg, Creb3l1, Lypd1*) were found to be upregulated. Six genes were validated on quantitative polymerase chain reaction and subsequently, Amphiregulin (*Areg*) was selected (based on log2 fold change) for further experiments to unravel its involvement in LID. Areg LV_shRNA was used to knock down *Areg* to explore its therapeutic role in the LID model.

**Results:**

Western blotting and immunofluorescence results show that AREG is significantly expressed in the LID group relative to the control. Dyskinetic movements in LID mice were alleviated by *Areg* knockdown, and the protein expression of delta FOSB, the commonly attributable protein in LID, was decreased. Moreover, *Areg* knockdown reduced the protein expression of P‐ERK. In order to ascertain whether the inhibition of the ERK pathway (a common pathway known to mediate levodopa‐induced dyskinesia) could also impede *Areg*, the animals were injected with an ERK inhibitor (PD98059). Afterward, the AIMs, AREG, and ERK protein expression were measured relative to the control group. A group treated with ERK inhibitor had a significant decrease of AREG and phosphorylated ERK protein expression relative to the control group.

**Conclusion:**

Taken together, our results indicate unequivocal involvement of *Areg* in levodopa‐induced dyskinesia, thus a target for therapy development.

## Graphical abstract

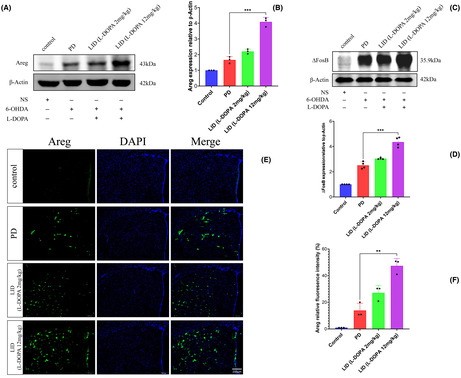

## 1 INTRODUCTION

Parkinson's disease (often known simply as PD) is one of the most prominent kinds of neurodegenerative disease with unexpected intricacies. The neuropathology of this neurodegenerative disease is very well characterized; however, the etiology of the disease is still unknown, which makes it challenging to target therapy. Levodopa (L‐DOPA) is reputed to be the most effective drug for improving movement impairments in Parkinson's disease. By replacing the dopaminergic neurons in the substantia nigra that were lost due to degeneration, this treatment can improve motor symptoms.^1^ Unfortunately, after using L‐DOPA for a while, most persons with PD develop abnormal involuntary movements (AIMs), often known as L‐DOPA‐induced dyskinesia (LID).^2^, ^3^ Dyskinesia is a side effect of L‐DOPA treatment that worsens with continued use, negating the drug's therapeutic effects. The mechanisms underpinning levodopa‐induced motor swings and dyskinesia remain controversial.^4^ One acknowledged assertion for this phenomenon is the engagement of both presynaptic and postsynaptic pathways, which ultimately result in non‐physiologic excitation of the pulsatile dopamine receptor, leading to a variety of dysfunctional neuronal behaviors.^5^, ^6^ Nonetheless, a luculent molecular encompassment of levodopa‐induced dyskinesia is perplexing, and indeed studies demonstrate that even its evolution is quite capricious. Understanding the molecular signatures of levodopa‐induced dyskinesia could pave the way to anti‐dyskinetic novel therapy development to halt the impact of dyskinesia in Parkinson's disease. To unravel this, we used the Linear Models for Microarray Analysis (LIMMA) R tool from the Bioconductor project to analyze the microarray data set (GSE55096) from the gene expression omnibus (GEO) repository and identified the differentially expressed genes (DEGs). Twelve genes were found to be upregulated (*Nr4a2*, *Areg*, *Tinf2*, *Ptgs2*, *Pdlim1*, *Tes*, *Irf6*, *Tgfb1*, *Serpinb2*, *Lipg*, *Creb3l1*, and *Lypd1*). Subsequently, Amphiregulin (*Areg)* was selected (based on log2 fold change) for further experiments to unravel its involvement in LID. Western blotting and immunofluorescence results show that *Areg* is significantly expressed in the LID group relative to the control. Dyskinetic movements in LID mice were alleviated by *Areg* knockdown, and the protein expression of delta FOSB, the commonly attributable protein in LID, was decreased. In order to ascertain whether the inhibition of Extracellular‐signal‐regulated kinase (ERK) pathway (this pathway is commonly known to mediate the levodopa‐induced dyskinesia) could also impede *Areg*, the animals were injected with ERK inhibitor (PD98059). Afterward, the AIMs, AREG, and ERK protein expression were measured relative to the normal saline group. A group treated with ERK inhibitor had a significant decrease in AREG and P‐ERK protein expression relative to the normal saline group. Taken together, our results indicate an equivocal involvement of *Areg* in levodopa‐induced dyskinesia, thus a target for therapy development.

## 2 MATERIALS AND METHODS

### 2.1 Microarray data

The microarray expression profiling dataset GSE55096, deposited by Heiman et al., was retrieved from the Gene Expression Omnibus database (https://www.ncbi.nlm.nih.gov/geo/).^7^ The Gene Expression Omnibus database at the National Center for Biotechnology Information (NCBI) and Array Express at the European Bioinformatics Institute (EBI) are the two major public databases of microarray data. The GEO database has offered a robust platform for the field of bioinformatics, which has allowed researchers to investigate novel biomarkers for the diagnosis, therapy, and analysis of cancer prognosis.^8^ The original data was centered on the GPL1261 Affymetrix Mouse Genome 430 2.0 array platform. The experiment contained 77 samples consisting of 44 subjects of direct spiny projection neurons (dSPNs) (direct pathway) and 33 indirect spiny projection neurons (iSPNs) (indirect pathway). The mouse model used in this data set is validated and it uses the green florescence to tag these pathways (Refer to Supporting Information of the metadata). This was further categorized into 6‐hydroxydopamine (6‐OHDA) Parkinson's disease model or mock lesion (Ascorbate). The 6‐OHDA was further subdivided into chronic high levodopa, chronic low levodopa, or chronic saline.

### 2.2 Differential expression analysis

Differential expression analysis was performed using the online analysis tool GEO2R (https://www.ncbi.nlm.nih.gov/geo/geo2r). GEO2R is an online tool for identifying differentially expressed molecules across various experimental conditions.^9^ It performs comparisons on original submitter‐supplied processed data tables using the GEO query and Limma R packages from the Bioconductor project.^10^ Bioconductor is an open‐source software project based on the R programming language that provides tools for the analysis of high‐throughput genomic data.^11^ The data from three groups (Chronic high levodopa, chronic low levodopa, and chronic saline) were first visualized in Uniform Manifold Approximation and Projection plot (UMAP). Only the direct pathway yielded clear differences between these three groups on the UMAP plot. Hence, our further analysis and validation were performed on the direct pathway. After differential gene expression analysis, each sample's genes fulfilling the following conditions were retained: (1) Upregulated (*p*‐adjusted value ˂0.05, log2 fold change ≥1.5), (2) Downregulated (*p*‐adjusted value ˂0.05, log2 fold change ≤ −1.5). The volcano plot of each sample against the other was plotted in the GEO2R tool. A web‐based tool Venny 2.1 (http://bioinfogp.cnb.csic.es/tools/venny/index.html) was used to construct a Venn diagram. Heml tool was used to generate a heatmap of the DEGs.

### 2.3 Animals

For the purpose of validating those genes obtained after analysis, male and female mice (C57BL6/6J) were enquired from the animal center of Xuzhou Medical University and maintained in a regulated environment with access to food and water. The temperature was set at (23 ± 2°C) and the lighting was controlled (12 h light; 12 h dark). The animal treatment complied with the ethics set forth by the Animal Center of Xuzhou Medical University.

### 2.4 6‐OHDA Parkinson's disease mouse model

6‐OHDA (MedChemExpress) was stereotaxically injected into the left striatum in order to generate Parkinson's disease in a mouse model. In a nutshell, mice were put onto a stereotactic frame after being given intraperitoneally sodium pentobarbital anesthesia (45 mg/kg; Sigma, St. Louis, MO, USA). 6‐OHDA was dissolved at a concentration of 2 μg/μL saline in 0.2% ascorbic acid and 2 μL was administered into the left striatum (0.5 mm anterior‐posterior, 1.5 mm lateral, 3.5 mm ventral to the dural surface) using a Hamilton syringe. The control model was administered 2 μL of normal saline into the same coordinates of the striatum at the pace of 0.5 μL/min. After the injection, the needle remained in place for 5 min before being retracted.

### 2.5 Open field test

Open field test was performed as we previously elaborated.^12^ In a nutshell, mice were first injected with apomorphine (0.5 mg/kg, intraperitoneally). Subsequently, animals' rotation and distance traveled were digitally recorded and analyzed using the ANY‐maze video‐tracking system (Muromachi Kikai, Tokyo, Japan).

### 2.6 Stereotaxic injection of Areg LV_shRNA

Santa Cruz Biotechnology (Dallas, TX, USA) supplied the Areg LV shRNA viral stock, which had 1 × 10^6^ infectious viral units in 200 μL. These lentiviral particles were handed as viral particles that were ready to be used for gene silencing. The particles carried 3 to 5 expression constructs, of which each included a target‐specific 19–25 nucleotide sequence with a hairpin to produce a shRNA intended to inhibit gene expression. More information on how the Areg LV_shRNA plasmid was constructed may be found in Additional Figure 1. Next, mice were put onto a stereotactic frame after being given intraperitoneally sodium pentobarbital anesthesia (45 mg/kg; Sigma, St. Louis, MO, USA). The same striatum coordinates (0.5 mm anterior‐posterior, 1.5 mm lateral, 3.5 mm ventral to the dural surface) were used to inject 5 μL of either Areg LV_shRNA or Areg LV_Scramble, referred to here as the normal control (NC). Three weeks post‐viral infusion, *Areg* knockdown was confirmed by quantitative polymerase chain reaction.

**FIGURE 1:**
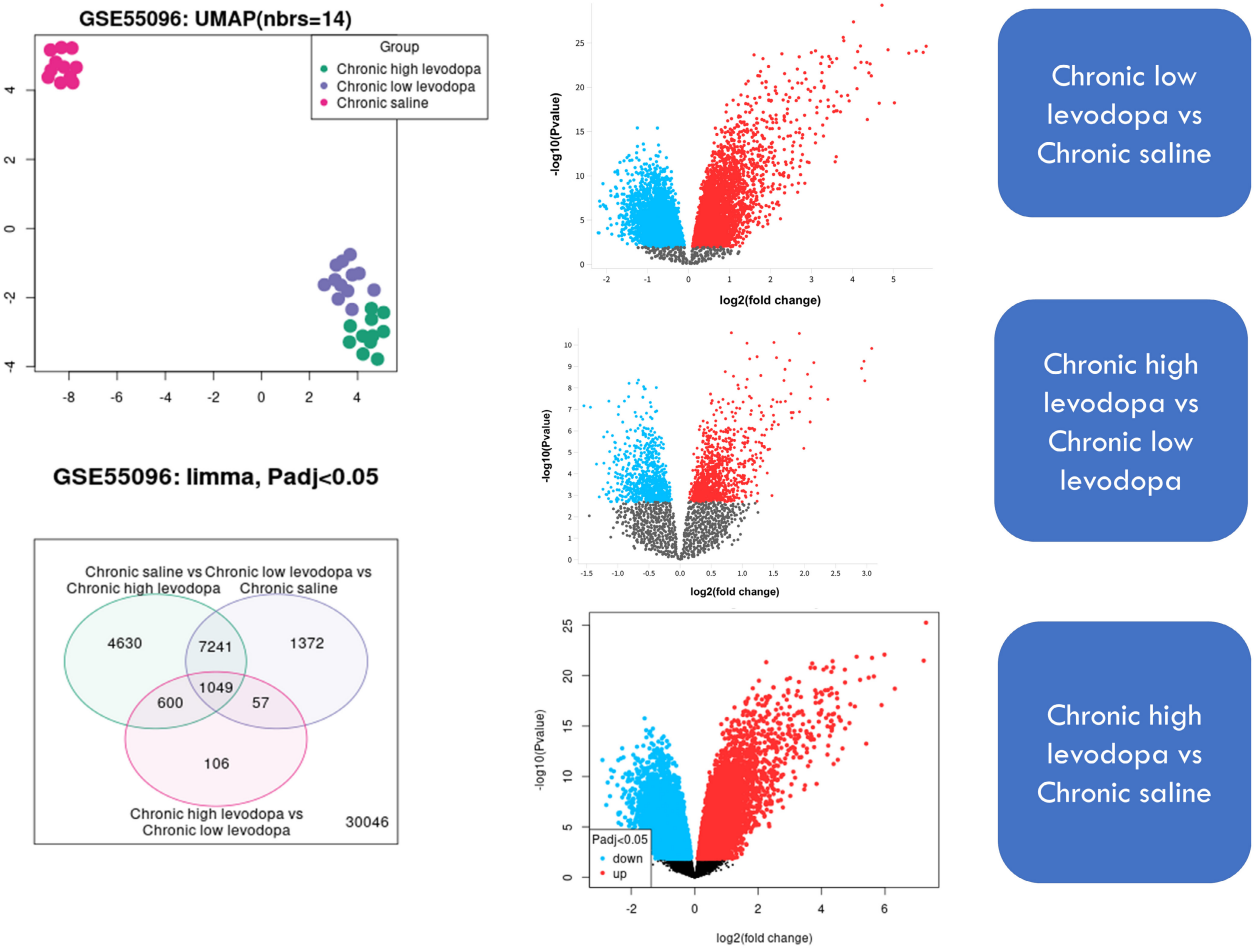
Analysis of the microarray data set (GSE55096) using Linear Models for Microarray Analysis (LIMMA). A clear discrimination appears between the chronic saline and chronic levodopa (Low/High). A further analysis of each group against the other indicates the upregulated genes (red color) and the downregulated genes (blue color). The condition used here was *p*‐adjusted ˂0.05. A total of 1049 genes were found to participate in the three treatment groups. UMAP: Uniform Manifold Approximation and Projection plot, Padj: *p*‐adjusted value.

### 2.7 Levodopa injection and AIMS observation

Animals that were successfully modeled for Parkinson's disease were divided into three groups. The first group was administered an intraperitoneal injection of a mixed solution of levodopa (12 mg/kg, Cat. No. HY‐N0304, MedChemExpress, Shanghai, China) and Benserazide hydrochloride (10 mg/kg, Cat. No. HY‐B0404A, MedChemExpress, Shanghai, China). The second group received a lower dose (L‐DOPA 2 mg/kg, Cat. No. HY‐N0304, MedChemExpress, Shanghai, China) and Benserazide hydrochloride (2 mg/kg, Cat. No. HY‐B0404A, MedChemExpress, Shanghai, China). The third group was given a placebo of 0.9% NaCl. After levodopa injection, the mice were monitored for 25 min daily, and every third day, their aberrant movements (AIMS) were recorded.^13^, ^14^ Fourteen days later, on day 14, we observed for 2 h and rated the AIMS. Two observers who were naïve to the treatment documented and classified the severity and nature of dyskinetic behaviors. Animals were euthanized 30 min following the final levodopa administration in all experiments.

### 2.8 RNA extraction and real‐time quantitative polymerase chain reaction

Mice were executed by cervical dislocation, and the brains were quickly removed and dropped into ice‐cold saline. On an icy plastic surface, the left striatum and midbrain were then dissected. Quantitative polymerase chain reaction was performed using the previously reported approach.^12^ Samples were quickly minced in 1 mL of TRIzol. The samples were then put into a 1.5 mL centrifuge tube, to which 200 μL of chloroform was added, properly mixed, and incubated on ice for 10 min before centrifuging for 15 min at 12,000× *g*, 4°C. After being centrifuged at 12,000× *g* for 10 min at 4°C, the supernatant was transferred to a 1.5‐mL tube free of RNAse, gently mixed with 0.5 mL of isopropyl alcohol, and incubated at room temperature for 10 min. The RNA precipitate was centrifuged at 7500× *g* for 5 min at 4°C after being washed with 1 mL of 75% ethanol and the supernatant was discarded. The centrifuge tube was turned upside down, dried on a spotless work surface, and then the RNA was dissolved in 40 μL of DEPC‐treated water. A spectrophotometer (Thermo, Boston, MA, USA) was used to measure the optical density of the RNA sample at 260 and 280 nm in order to calculate its concentration. After balancing the mRNA concentration, PrimeScriptTM, RT Master Mix was used to reverse‐transcribe the mRNA into cDNA. The temperature settings for the PCR were 25°C for 10 min, 42°C for 30 min, and 85°C for 5 min. The primers used are indicated in Table 1. Glyceraldehyde‐3‐phosphate dehydrogenase (*Gapdh*) mRNA was used as an internal control, and the results were analyzed with a delta–delta Ct method.

**TABLE 1:** List of primers used in qPCR assay.

Primer	Nucleotide sequence 5′‐3′	Amplicon size (bp)
*Areg‐Foward*	GGTCTTAGGCTCAGGCCATTA	137
*Reverse*	CGCTTATGGTGGAAACCTCTC	137
*Nr4a2‐Foward*	GTGTTCAGGCGCAGTATGG	153
*Reverse*	TGGCAGTAATTTCAGTGTTGGT	153
*Tinf2‐Foward*	TGCCCTGAAGCATCACTTCC	166
*Reverse*	GGCAACTAGAAAGGATTCCCC	166
*Pdlim1‐Foward*	TCGATGGGGAAGATACCAGCA	102
*Reverse*	TCTGTTCAGACCTGGATACTGTG	102
*Ptgs2‐Foward*	TTCAACACACTCTATCACTGGC	271
*Reverse*	AGAAGCGTTTGCGGTACTCAT	271
*Tes‐Foward*	AGCCCCCTGTCTAAAATGCAA	167
*Reverse*	GGGTGGTGTACTTAGTGTCCTC	167
*Gapdh‐Foward*	AGGTCGGTGTGAACGGATTTG	123
*Reverse*	TGTAGACCATGTAGTTGAGGTCA	123

### 2.9 Western blot

Striatum and ventral midbrain proteins were extracted as described in our earlier work.^12^ In essence, animals were sacrificed, their brains were swiftly removed, and the ipsilateral ventral midbrain was dissected, instantly stored on dry ice for immediate use, and then placed in a refrigerator set at −80°C for later use. In a 100:1 mixture of Phenylmethylsulfonyl fluoride (PMSF) and RIPA buffer (Beyotime, China), tissues were homogenized (RIPA: PMSF). Homogenized proteins were centrifuged at 12,000× *g* for 30 min at 4°C. Using the Bicinchoninic Acid Protein Assay Kit (Beyotime, China), the protein concentrations were determined. 20 μg of proteins were separated by 10% Sodium Dodecyl Sulfate–Polyacrylamide Gel Electrophoresis (SDS‐PAGE) and electrotransferred to nitrocellulose membranes for 1 h at 100 volts. We blocked the membrane with 5% skim milk in Tris‐buffered saline for 2 h before overnight incubation at 4°C with the primary antibodies listed in Table 2. The membranes were treated with the corresponding secondary antibodies the following day (Table 2). After blotting, an image analyzer was used to scan and review the filter bands (odyssey scanner‐ USA). Three western blotting repeats were examined at least, and the results were displayed in box plots with data points.

**TABLE 2:** List of antibodies used in western blotting assay.

Antibody	Catalog number	RRID number	Company
TH	25859‐1‐AP	AB_2716568	Proteintech
AREG	16036‐1‐AP	AB_2227602	Proteintech
Delta FOSB	Ab11959	AB_298732	Abcam
ERK	4695S	AB_390779	Cell Signaling Technology
P‐ERK	4370S	AB_2315112	Cell Signaling Technology
Beta Actin	81115‐1‐RR	AB_2923704	Proteintech
Goat anti‐rabbit	Ab205718	AB_2819160	Abcam
Goat anti‐mouse	Ab205719	AB_2755049	Abcam

### 2.10 Immunofluorescence staining

After anesthesia with pentobarbital (45 mg/kg) and heart perfusion (with 100 mL of PBS followed by 150 mL of cold 4% paraformaldehyde), the brains were collected and cryoprotected in a solution of 20% (for 24 h) and 30% (for 24 h) sucrose in 0.1 M phosphate buffer at 4°C. The striatum was cut into coronal sections that were 16 μm thick, and they were left to air dry for an entire day. Following that, the sections were blocked for 2 h with 0.1% Triton X‐100 and 10% normal goat serum mixed in 0.1 M PB. Sections were incubated with AREG antibody (rabbit, 1:200, Proteintech, Cat# 16036‐1‐AP) overnight at 4°C. The stained sections were properly cleaned before being incubated for 6 h at room temperature with Alexa Fluor 488‐conjugated AffiniPure goat antirabbit IgG (1:200, Jackson ImmunoResearch, Carlsbad, CA, USA, Cat# 111‐545‐003, RRID: AB 2338046). For dual immunofluorescence, sections were incubated overnight at 4°C with either a mixture of dopamine 1 receptor (D1R) (cat # 17934‐1‐AP, RRID: AB 10598308, Proteintech) and AREG (cat # sc‐74501, RRID: AB 2227602, Santa Cruz) antibodies or dopamine 2 receptor (D2R) (cat # 55084‐1‐AP, RRID: AB 10859941, Proteintech) and AREG. The sections were then appropriately stained with their respective counter‐secondary antibodies. Goat Anti‐Mouse IgG H&L (Alexa Fluor® 488) (cat # Ab150113, Abcam) and Goat Anti‐Rabbit IgG H&L (Alexa Fluor® 594) (cat # Ab150080, Abcam) were used as secondary antibodies. The nuclei were counterstained with 4′,6‐diamidino‐2‐phenylindole (DAPI) for 15 min. Using the fluorescence microscope, the degree of immunoreactivity was measured in comparison to the counterstain (DAPI).

### 2.11 Statistical analysis

The statistical analysis was performed using GraphPad Prism for Windows, Version 9.0.0 (GraphPad Software, San Diego, CA, USA). Groups were compared using Student's *t*‐test. Results are shown as a mean ± standard deviation (SD). At a significance level of *p* ˂ 0.05, the results were regarded as significant.

## 3 RESULTS

### 3.1 Analysis of the microarray data set (GSE55096) using LIMMA informs 12 differentially upregulated genes involved in levodopa‐induced dyskinesia

**FIGURE 2:**
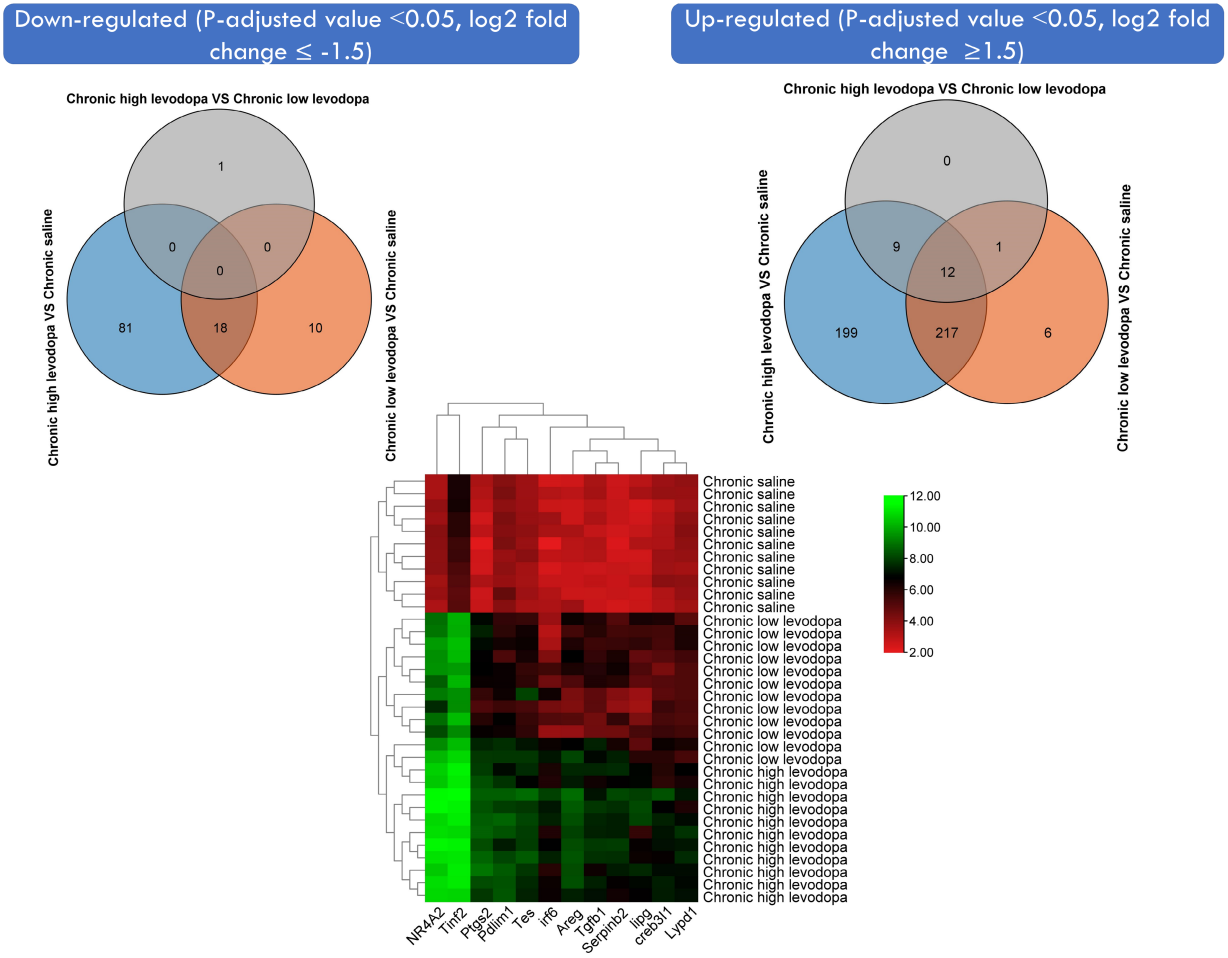
12 differentially upregulated genes involved in levodopa‐induced dyskinesia. The 1049 genes were further analyzed using more criteria. By utilizing the criteria [Downregulated (*p*‐adjusted value ˂0.05, log2 fold change ≤ −1.5)], [Upregulated (*p*‐adjusted value ˂0.05, log2 fold change ≥1.5)], we found that only 12 genes were upregulated and none were downregulated. The heatmap shows the Log2 values of Robust Multiarray Average (RMA) of each gene per group.

In order to ascertain the differentially expressed genes associated with levodopa‐induced dyskinesia, analysis of GSE55096 data set was done using the LIMMA package from Bioconductor. The data set included three different treatment groups (Chronic saline, Chronic low levodopa, and chronic high levodopa). The UMAP dimension reduction technique was used to explore the clusters. Analysis of each group against the other to reveal the upregulated and downregulated in the volcano plot is indicated in Figure 1. In total, 1049 significant genes (*p*adj ˂ 0.05) were found in both three groups. Subsequently, these genes were further analyzed using more criteria. Using the criteria [Downregulated (*p*‐adjusted value ˂0.05, log2 fold change ≤−1.5)], [Upregulated (*p*‐adjusted value ˂0.05, log2 fold change ≥1.5)], we found that only 12 genes were upregulated and none were downregulated. The heatmap shows the Log2 values of robust multiarray average (RMA) of each gene per group (Figure 2).

### 3.2 Differentially expressed genes validated for mice brain samples of levodopa‐induced dyskinesia are hyper‐expressed in the striatum but not in the midbrain

**FIGURE 3:**
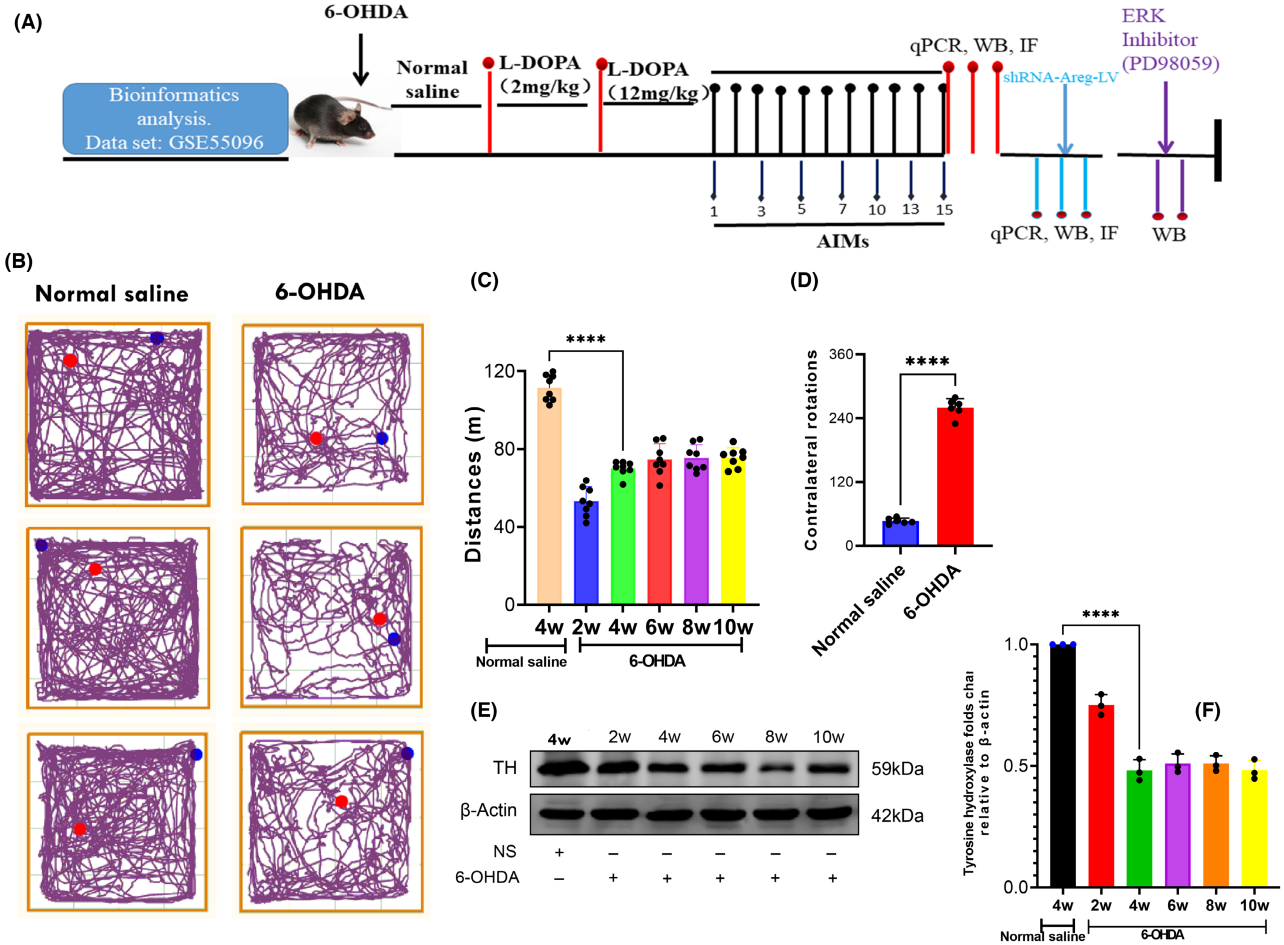
Schematic depictions of the experiment's flow and the induction of 6‐hydroxydopamine (6‐OHDA) Parkinson's disease mouse model. After a preliminary bioinformatics analysis, a Parkinson's disease model was generated, and groups were formed accordingly (A). The Parkinson's disease mouse model was induced and confirmed using the open field test (B). The distance traveled by an animal in the field (C), as well as the contralateral rotation (D) were analyzed. The western blot analysis revealed that the marker of dopaminergic neurons (Tyrosine hydroxylase) reduced significantly from the fourth week (E, F). The data are depicted as the mean ± SD (*n* = 8). Student's *t*‐test, **p < 0.01, ***p < 0.001. 6‐OHDA: 6‐Hydroxydopamine.

**FIGURE 4:**
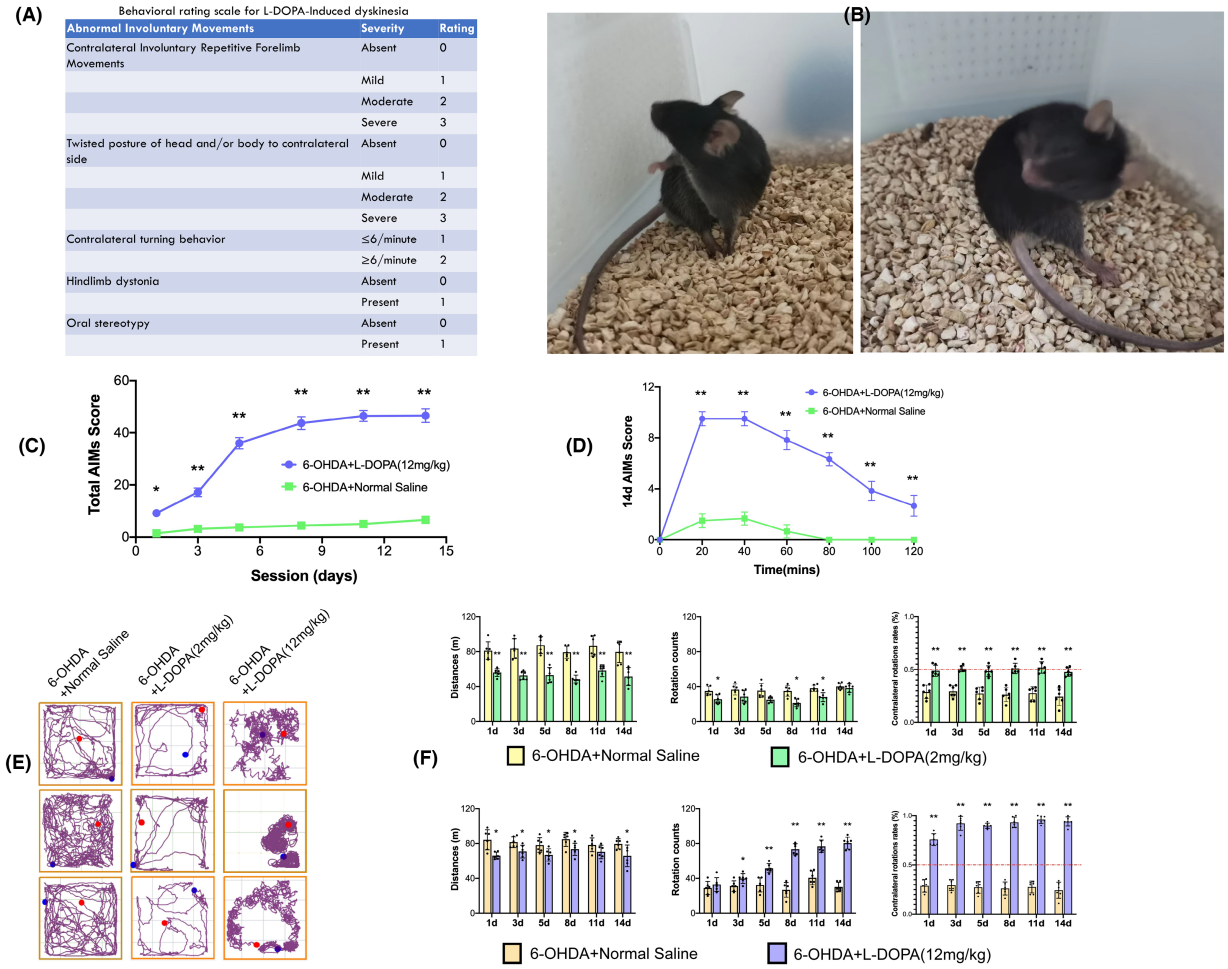
Observation and analysis of Abnormal Involuntary Movements (AIMS). Behavioral rating parameters for L‐DOPA‐Induced dyskinesia as indicated in (A) were used to rate the dyskinetic movements. A representative figure of the contralateral turning behavior is indicated in (B). The results indicate a significant abnormal movement in a group treated with levodopa compared to the normal saline group (C, D). Figure (D) shows an extended observation and recording (2 h). Figure (E) and (F) shows the open field behavior analysis after chronic levodopa injection. The data are depicted as the mean ± SD (*n* = 20). Student's *t*‐test, **p < 0.01.

**FIGURE 5:**
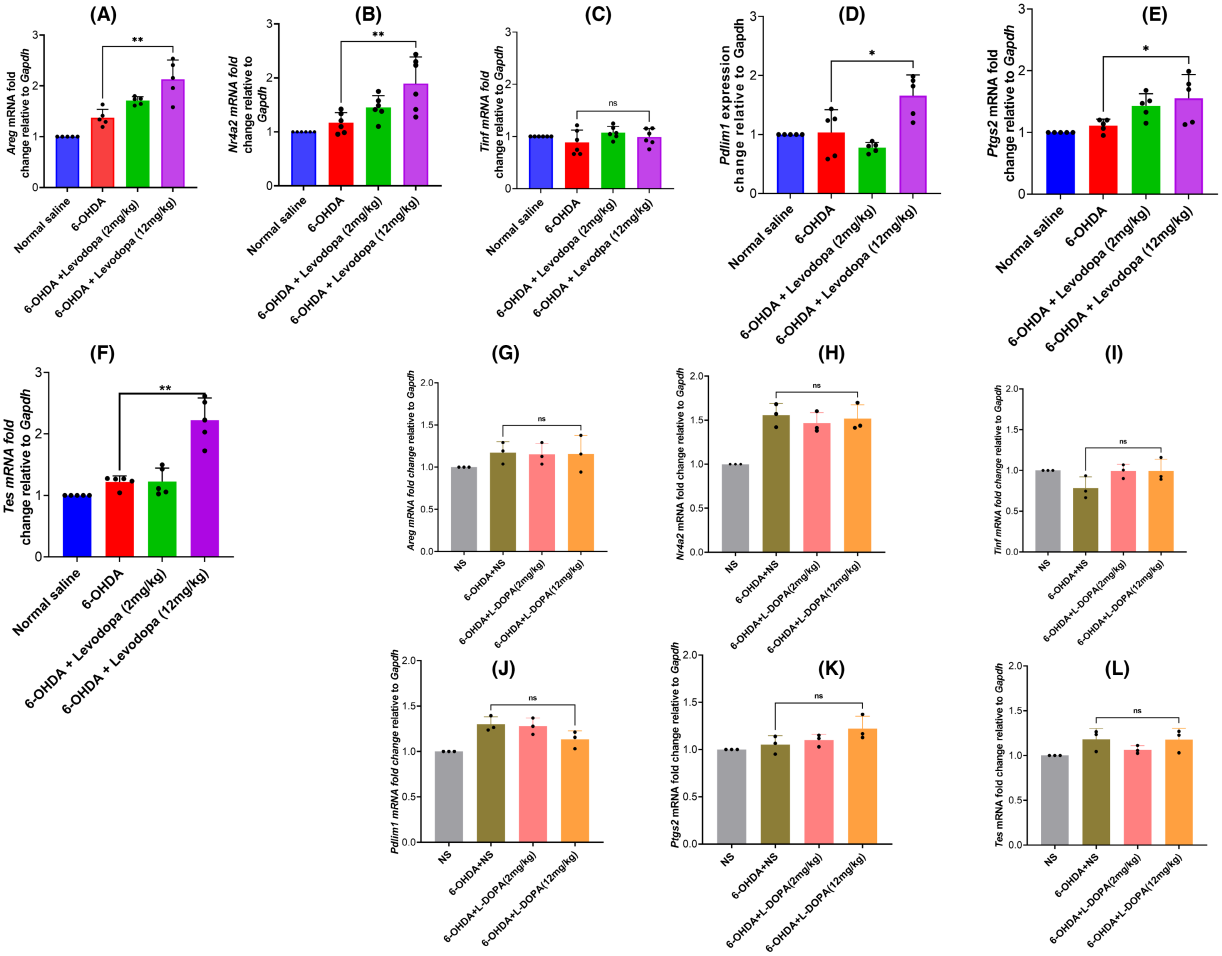
Differentially expressed genes validated for mice brain samples of levodopa‐induced dyskinesia are hyper‐expressed in the striatum but not in the midbrain. The striatum and midbrain were extracted and processed for quantitative polymerase chain reaction. The results show that these genes are significantly upregulated in the striatum (A–F) but not in the midbrain of levodopa‐induced dyskinetic mice (G–L). The data are depicted as the mean ± SD (*n* = 15). Student's *t*‐test, **p < 0.01.

First, we induced a Parkinson's disease mouse model by stereotaxically injecting 6‐hydroxydopamine into the striatum. Figure 3A indicates the schematic illustration of the study. The behavior test and western blot were done after 2,4,6,8, and 10 weeks post the injection. The open field test indicated in Figure 3B,C shows that mice injected with 6‐OHDA traveled a shorter distance (*p* = 0.0001, *t* = 15.09, *df* = 14) and rotated more contralaterally on apomorphine compared to those injected with normal saline (Figure 3D, *p* = 0.0001, *t* = 29.37, *df* = 10). The western blotting results indicate a reduced Tyrosine hydroxylase expression from the fourth‐week post 6‐OHDA injection (Figure 3E,F, *p* = 0.0001, *t* = 20.24, *df* = 4). This is to prove that the model was successful. Subsequently, LID was induced. To attain this, 4 weeks after 6‐OHDA injection, the animals were divided into 3 groups (Normal saline, Low dose of levodopa‐2 mg/kg, and high dose of levodopa‐12 mg/kgi). These 3 groups were intraperitoneally injected with either normal saline or levodopa (low/high) for 15 days daily. The AIMS and open field test were analyzed as indicated in Figure 4. The results indicate a significant abnormal movement in a group treated with levodopa compared to the normal saline group. A supporting video has also been provided (Supporting videos S1 and S2). Furthermore, 20 min after the last dose of levodopa, the striatum and midbrain were extracted and processed for a quantitative polymerase chain reaction. From the 12 differentially expressed genes, we selected 6 genes according to their log2 FC and the expression on the heatmap. *Areg, Nr4a2, Tinf2, Ptgs2, Pdlim1*, and *Tes*, were the genes validated. The results show that these genes are significantly upregulated in the striatum (Figure 5A–F) but not in the midbrain of levodopa‐induced dyskinetic mice (Figure 5G–L).

### 3.3 Amphiregulin is highly expressed in the levodopa‐induced dyskinesia 6‐OHDA Parkinson's disease mouse model

**FIGURE 6:**
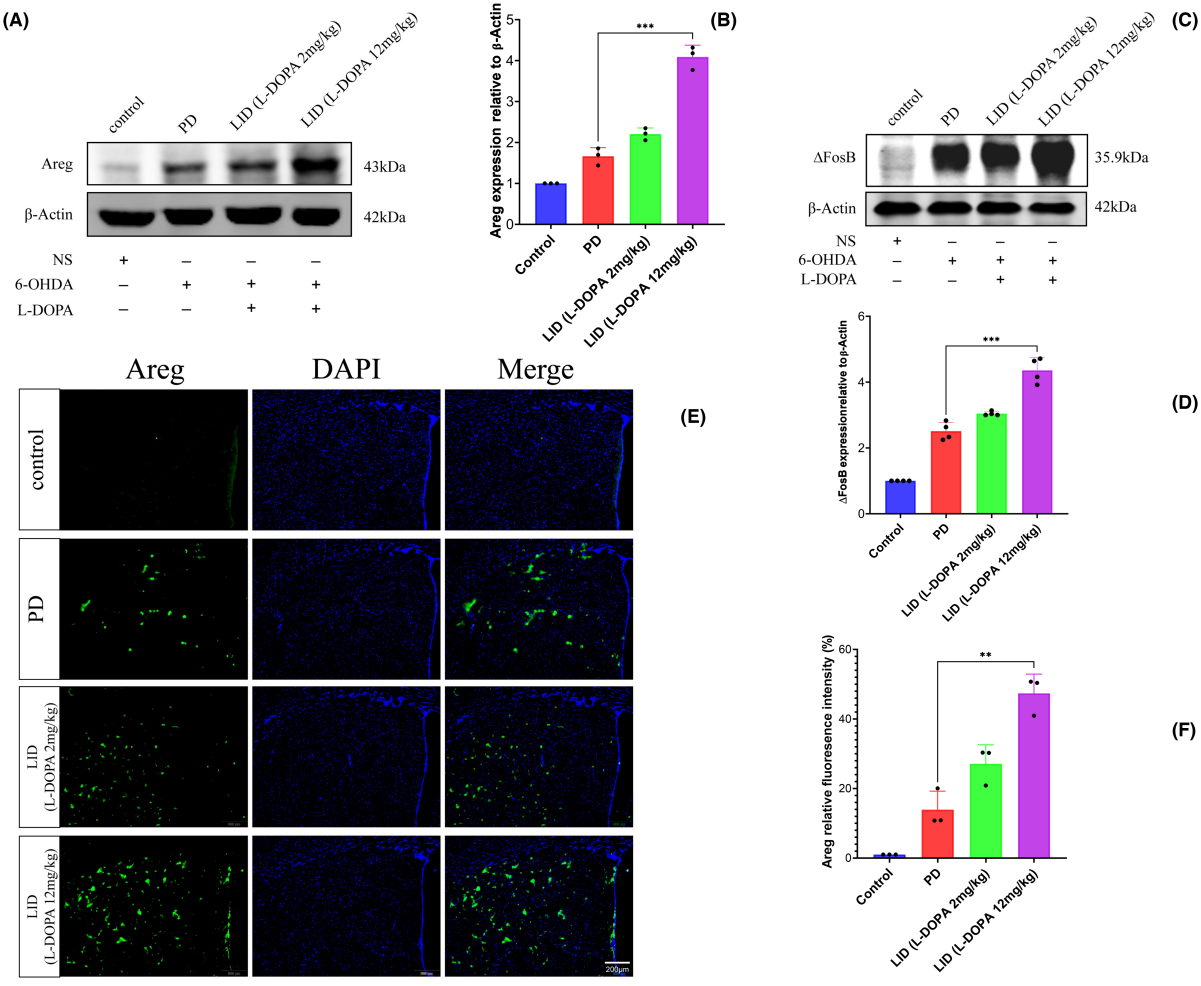
Amphiregulin (Areg) is highly expressed in the levodopa‐induced dyskinesia 6‐OHDA Parkinson's disease mouse model. The Parkinson's disease group treated with a daily 12 mg/kg of levodopa for 15 days developed dyskinetic movement. On western blotting, this group further showed a significant increase of Amphiregulin protein expression relative to the Parkinson's disease group injected with normal saline only (A, B). These results are also concordant with the known marker of levodopa‐induced dyskinesia (FosB) (C, D). On immunofluorescence, AREG‐positive neurons were remarkably activated in the 12 mg/kg levodopa‐induced dyskinesia group (E, F). The data are depicted as the mean ± SD (*n* = 8). Student's *t*‐test, **p < 0.01.

To further explore the protein expression of some of these differentially expressed genes, we chose to investigate Amphiregulin (*Areg)* because of its central role in inflammation.^15^ Neuroinflammation is thought to be involved in the pathogenesis of levodopa‐induced dyskinesia.^16^, ^17^ The Parkinson's disease group treated with a daily 12 mg/kg of levodopa for 15 days developed dyskinetic movement. On western blotting, this group further showed a significant increase of Amphiregulin protein expression relative to the Parkinson's disease group injected with normal saline only (*p* = 0.0003, *t* = 11.85, *df* = 4, Figure 6A,B). These results are also concordant with the known marker of levodopa‐induced dyskinesia (FosB) (*p* = 0.0002, *t* = 7.863, *df* = 6, Figure 6C,D). On immunofluorescence, AREG‐positive neurons were remarkably activated in the 12 mg/kg levodopa‐induced dyskinesia group (Figure 6E,F).

### 3.4 AREG+ are highly expressed in D1R but not in D2R

**FIGURE 7:**
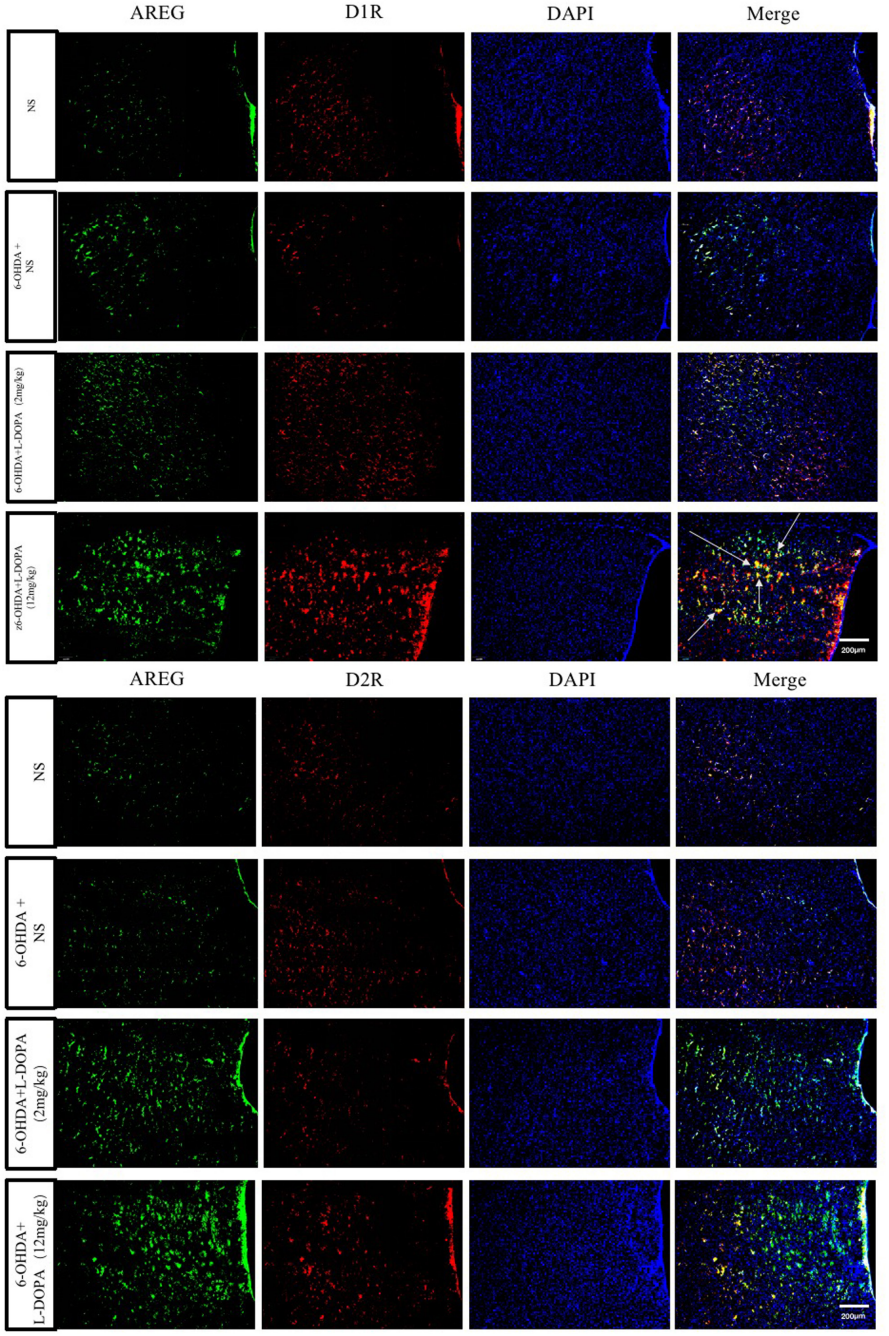
AREG+ are highly expressed in dopamine 1 receptor (D1R) but not in dopamine 2 receptor (D2R). Using the double immunofluorescence to explore the co‐occurrence of AREG+/D1R+ or AREG+/D2R+, the findings indicate that AREG+ are markedly co‐expressed with D1R, but not D2R (Arrows in the upper figure). (*n* = 3).

Next, we sought to determine the dopamine receptor family to which AREG‐positive neurons belong. For this case, double immunofluorescence was used to explore the co‐occurrence of AREG+/D1R+ or AREG+/D2R+. Previous studies have demonstrated that both D1R and D2R are responsible for levodopa‐induced dyskinesia. Using a D1R agonist^18^ or a D2R agonist^19^ induced dyskinetic movements highly similar to those of LID. Our results show that AREG+ are markedly co‐expressed with D1R, but not D2R (Arrows in Figure 7). This suggests that D1R is indispensable for AREG in LID.

### 3.5 Knocking down Amphiregulin significantly decreases the AIMs owing to chronic levodopa treatment

**FIGURE 8:**
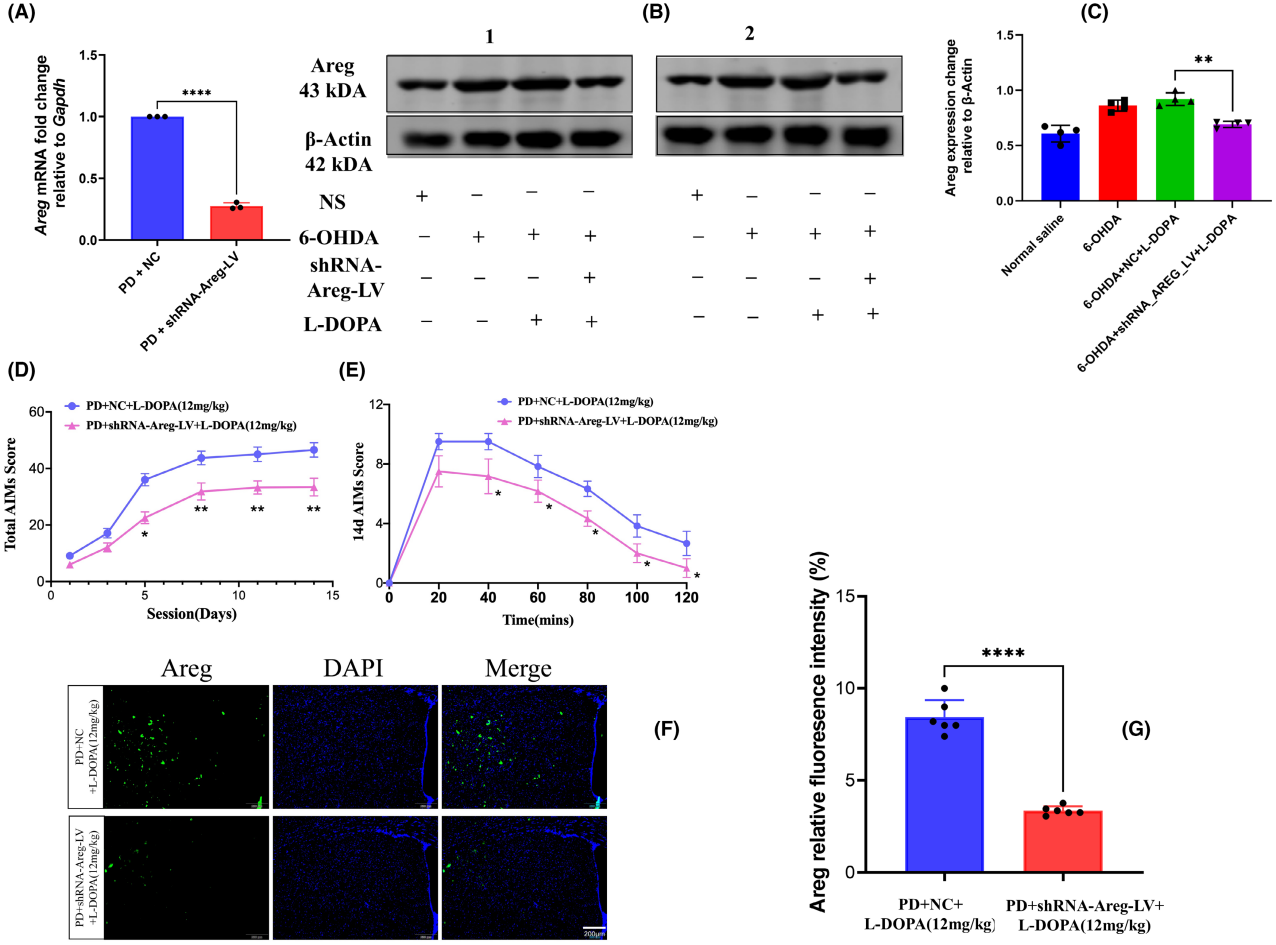
Knocking down Amphiregulin significantly decreases the abnormal involuntary movements owing to chronic levodopa treatment. The results indicate that the *Areg* knockdown group had a significant decrease in *Areg* mRNA (A). When the levodopa was injected in order to cause dyskinesia, the *Areg* knocked down group showed an inhibited *Areg* protein expression (B, C), and consequently a significant decrease in total abnormal involuntary movements score is relative to the scramble injected group (D, E). On immunofluorescence, AREG‐positive neurons decreased considerably in a knockdown group compared to the scramble‐injected (F, G). The data are depicted as the mean ± SD (*n* = 10). Student's *t*‐test, **p < 0.01, ***p < 0.001.

Next, we sought to explore whether knocking down of Amphiregulin prior to levodopa treatment could decrease the AIMs. Four weeks after inducing Parkinsonism in an animal model, the lentiviral particles carrying *Areg* short hairpin RNA (*Areg_shRNA*) were injected through the same brain coordinates which were used to inject 6‐OHDA. Three weeks later, quantitative polymerase chain reaction was done to confirm the decrease in *Areg* after the knockdown. Subsequently, levodopa (12 mg/kg) was injected daily for 15 days. The AIMs were assessed every 2 days. The results indicate that the *Areg* knockdown group had a significant decrease in *Areg* mRNA (*p* = 0.0001, *t* = 47.09, *df* = 4, Figure 8A, Supporting video S3). When the levodopa was injected in order to cause dyskinesia, the *Areg* knocked down group showed an inhibited *Areg* protein expression (p = 0.0058, *t* = 7.082, *df* = 3 Figure 8B,C) and consequently a significant decrease in total AIMs score is relative to the scramble injected group (Figure 8D,E). On immunofluorescence, AREG‐positive neurons decreased considerably in a knockdown group compared to the scramble‐injected (Figure 8F,G).

### 3.6 Inhibition of the ERK pathway reduces the expression of Amphiregulin

**FIGURE 9:**
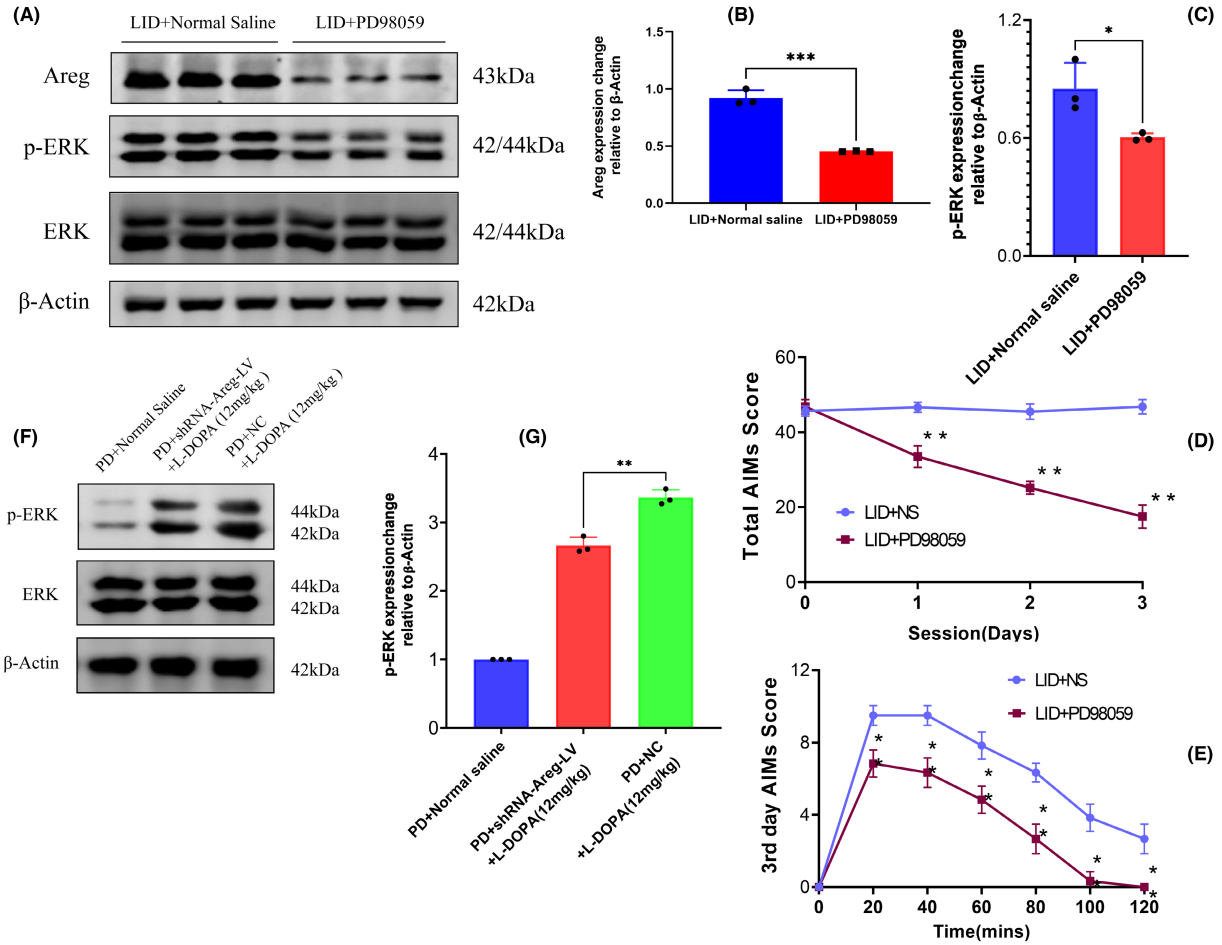
Inhibition of the Extracellular‐signal‐regulated kinase (ERK) pathway reduces the expression of Amphiregulin. A group treated with ERK inhibitor had a significant decrease of AREG and phosphorylated ERK protein expression relative to the control group (A–C). On AIMS analysis, the results show that the abnormal involuntary movements decreased significantly in a group injected with ERK inhibitor compared to that of normal saline (D). (E) indicates an extended observation and recording (2 hours) on the third day. In a manner similar to this, when we checked the expression of ERK in animals with *Areg* knockdown, we found that phosphorylated ERK reduced dramatically compared to the group injected with the scrambled virus (F, G). The data are depicted as the mean ± SD (*n* = 10). Student's *t*‐test, **p < 0.01.

ERK pathway has been undoubtedly reported to mediate levodopa‐induced dyskinesia. However, whether the inhibition of this pathway could also impede *Areg* which we report here as a crucial gene that facilitates dyskinetic movements secondary to chronic levodopa treatment remains to be elucidated. To this end, dyskinesia was first induced by injecting levodopa for 15 days. On the 15th day, animals were divided into two groups. The first group of dyskinetic animals (control) received normal saline 1 h before the next levodopa injection (Normal saline + LID). The second group of dyskinetic animals received ERK inhibitor (PD98059) 1 h before the next levodopa injection. Subsequently, the AIMs were observed and recorded. This process was repeated for four more days, making a total of 5 days for ERK inhibition and 19 days for levodopa injection. The results indicate that a group treated with ERK inhibitor had a significant decrease of AREG and phosphorylated ERK protein expression relative to the control group (*p* = 0.0003, *t* = 11.82, *df* = 4) Figure 9A–C. On AIMS analysis, the results show that the AIMs decreased significantly in a group injected with ERK inhibitor compared to that of normal saline (Figure 9D). Figure 9E indicates an extended observation and recording (2 hours) on the third day. In a manner similar to this, when we checked the expression of ERK in animals with *Areg* knockdown, we found that phosphorylated ERK reduced dramatically compared to the group injected with the scrambled virus (Figure 9F,G).

## 4 DISCUSSION

Exploring the possible causes of LID is critical in this dispensation where L‐DOPA still serves as the mainstay for Parkinson's disease treatment. The emergence of dyskinesia after several weeks or months of L‐DOPA treatment has confounded the understanding of its pathophysiology. There have been ramified attempts to uncover how L‐DOPA induces dyskinesia. Here, we started by analyzing the data set that was published on the gene expression omnibus repository. We used the criteria (*p*‐adjusted value ˂0.05, log2 fold change ≥1.5) or (*p*‐adjusted value ˂0.05, log2 fold change ≤−1.5) in establishing the upregulated and downregulated genes, respectively. Microarray data sets have long been valuable in identifying genes involved in cancer^20^ and neuroinflammation.^21^, ^22^ Our findings revealed 12 differently upregulated genes.

The meta‐data^7^ used in this study included low‐dose (2 mg/kg) and high‐dose (6 mg/kg) L‐DOPA. The rationale for utilizing two distinct doses (low/high) was to ascertain whether the dyskinetic movements and gene expression are dose‐dependent. This is because each previous study had its unique dose. For example, Wang et al. utilized 6 mg/kg,^23^, ^24^ Fahimi and Jahromy used 50 mg/kg,^25^ Cantuti‐Castelvetri and colleagues used 75 mg/kg,^14^ and Nicholas used 200 mg/kg.^26^ To ensure that we capture genes that participated in both dosages, we used a Venny diagram tool (https://bioinfogp.cnb.csic.es/tools/venny/index.html) to integrate the results from all groups. This means that the 12 obtained genes are expressed in both dosages. In the present study, we administered 2 and 12 mg/kg L‐DOPA, and the results indicate that both doses induce dyskinetic movements and gene expression alterations, but the larger dose has a greater effect.

It is worth noting that, of the 12 genes on our list, *Nurr1* has recently been linked to LID, and upregulation of *Nurr1* in the striatum perpetuates dyskinetic actions in a LID model.^27^ Our exhaustive search revealed no evidence of the genes *Tinf2*, *Ptgs2*, *Pdlim1*, and *Tes* being involved in neurodegenerative diseases. In fact, no neuronal‐related studies have been reported on these genes, according to the public repository platform https://platform.opentargets.org/ (Accessed in February 2023) which annotates evidence relevant to targets, diseases, phenotypes, and medications.^28^ This sparks a debate about the role of these genes in levodopa‐induced dyskinetic movements.

Our results show that *Areg, Nr4a2(Nurr1), Tinf2, Ptgs2, Pdlim1*, and *Tes* are hyper‐expressed in the striatum but not the midbrain. According to the existing literature, LID‐associated abnormal activity and molecular disruptions have been reported in various brain areas. Early investigations, for example, suggest that the primary motor cortex is involved in LID‐related dyskinetic movements.^29^, ^30^, ^31^ Furthermore, gene expression changes in the primary somatosensory cortex^32^ indicate maladaptive neuroplasticity after long‐term levodopa administration. Our findings are in line with previous literature, which shows that FOSB is dramatically expressed in the striatum of a LID model.^33^, ^34^ The hypothesis that an increase in dopamine release after L‐DOPA injection leads first to unusual changes in the striatum, a brain region that contains dopaminergic fibers from midbrain dopaminergic neurons, could explain why *Areg*, *Nr4a2*, *Tinf2*, *Ptgs2*, *Pdlim1*, and *Tes* are expressed in the striatum but not the midbrain.^35^ Notably, *Areg*, *Nr4a2*, and *Ptgs2* expression tended to increase in a comparable manner in both low and high doses of L‐DOPA, whilst *Pdlim1* and *Tes* expression were much lower in low‐dose L‐DOPA. This could be due to variances in the cells that express these genes and their responses to the dosage administered.

We chose to investigate Amphiregulin's involvement in LID because of the available evidence that *Areg* plays an important role in inflammation.^15^ Neuroinflammation is thought to be involved in the pathogenesis of levodopa‐induced dyskinesia.^16^, ^17^ Our findings show that *Areg* is pervasive in the levodopa‐induced dyskinesia of a 6‐OHDA Parkinson's disease mouse model. In concordance with this study, a prior investigation by Sellnow et al. found a similar outcome when they investigated the role of *Nurr1* in LID. They found that even in resistant subjects, ectopic stimulation of striatal Nurr1 can induce LID behavior and accompanying neuropathology.^27^ Our results indicate that AREG+ are markedly co‐expressed with D1R but not D2R. In contrast, a study by Solis et al. demonstrated that deleting D3R reduced the expression of LID markers such as FOSB.^36^ In our study, D3R was not explored. Nonetheless, this highlights the need for further investigation of AREG+ in other dopamine receptors.

The present study shows that knocking down Amphiregulin significantly decreases the AIMs owing to chronic levodopa treatment. In cell and molecular biology research, gene knockdown is a well‐established, cutting‐edge technique that is frequently employed to ascertain a gene's function. Gene knockdown is also employed therapeutically in addition to fundamental studies on gene function.^37^ Patisiran and Givosiran are good instances of gene knockdown‐based treatments that are now being used in clinical settings. Givosiran and Patisiran have received FDA approval for the treatment of people with acute hepatic porphyria and hereditary amyloidogenic transthyretin amyloidosis with polyneuropathy, respectively.^38^ Both the medications Patisiran and Givosiran contain siRNA, which lowers the expression of particular disease‐causing proteins. As a potential new therapeutic target for the treatment of levodopa‐induced dyskinesia, *Areg* knockdown may be of interest.

Myriad cellular responses are triggered by AREG signaling, and MAPK pathways are important to their execution. For instance, Lu et al. demonstrated that AREG stimulates hair regeneration of skin‐derived precursors through MAPK pathways.^39^ Similarly, AREG was discovered to activate the ERK MAPK in dental pulp stem cells, which in turn promoted their differentiation into odontoblasts.^40^ ERK–MAPK pathway has been undoubtedly reported to mediate the levodopa‐induced dyskinesia. Forced activation of dopamine receptor 1 (D1R), as reported by Picconi et al.,^41^ leads to the phosphorylation of ERK, a key component of LID.^41^ In a similar study, both ERK phosphorylation and FosB expression increased after prolonged L‐DOPA treatment.^42^ It appears that the increases in ERK activation and FosB expression are specific to the exquisitely sensitive reactivity to L‐DOPA induced by the dopaminergic lesion, as neither acute nor chronic L‐DOPA administration boost ERK phosphorylation in the dopamine‐intact striatum.^42^ The current findings show that inhibiting this route reduces Amphiregulin expression, which we establish as a key player gene in LID.

In conclusion, our findings suggest that *Areg* plays a conspicuous role in levodopa‐induced dyskinesia, making it a potential therapeutic target.

## AUTHOR CONTRIBUTIONS

In communication with Dianshuai Gao, Piniel Alphayo Kambey conceptualized and designed the study, conducted the analysis, and wrote the paper. Wen Ya Liu was responsible for conducting experiments and gathering data. Jiao Wu and Chuanxi Tang helped with the data collection. Wokuheleza Buberwa, Adonira Saro, and Alphonce M. K. Nyalali helped in data analysis. All authors reviewed the final manuscript and gave their approval.

## FUNDING INFORMATION

This project was funded by National Natural Science Research Funds of China (Grant No. 81971006).

## CONFLICT OF INTEREST STATEMENT

The authors claim they have no conflicting interests.

## Supporting information

Additional figure 1.

Additional figure 2.

Supporting video 1.

Supporting video 2.

Supporting video 3.

## Data Availability

Data that were gathered or evaluated during the course of the investigation are included in this article. The data that support the findings of this study are available in Gene Expression Omnibus (GEO) at https://www.ncbi.nlm.nih.gov/geo/query/acc.cgi?acc=GSE55096, reference number GSE55096. These data were derived from the following resources available in the public domain: GEO, https://www.ncbi.nlm.nih.gov/geo/query/acc.cgi?acc=GSE55096.
