# Supplementary figures and images for: Amphiregulin blockade decreases the levodopa‐induced dyskinesia in a 6‐hydroxydopamine Parkinson's disease mouse model
Source: CNS Neurosci Ther. 2023 Apr 26;29(10):2925–39. doi: 10.1111/cns.14229 (PMC10493657; doi:10.1111/cns.14229)

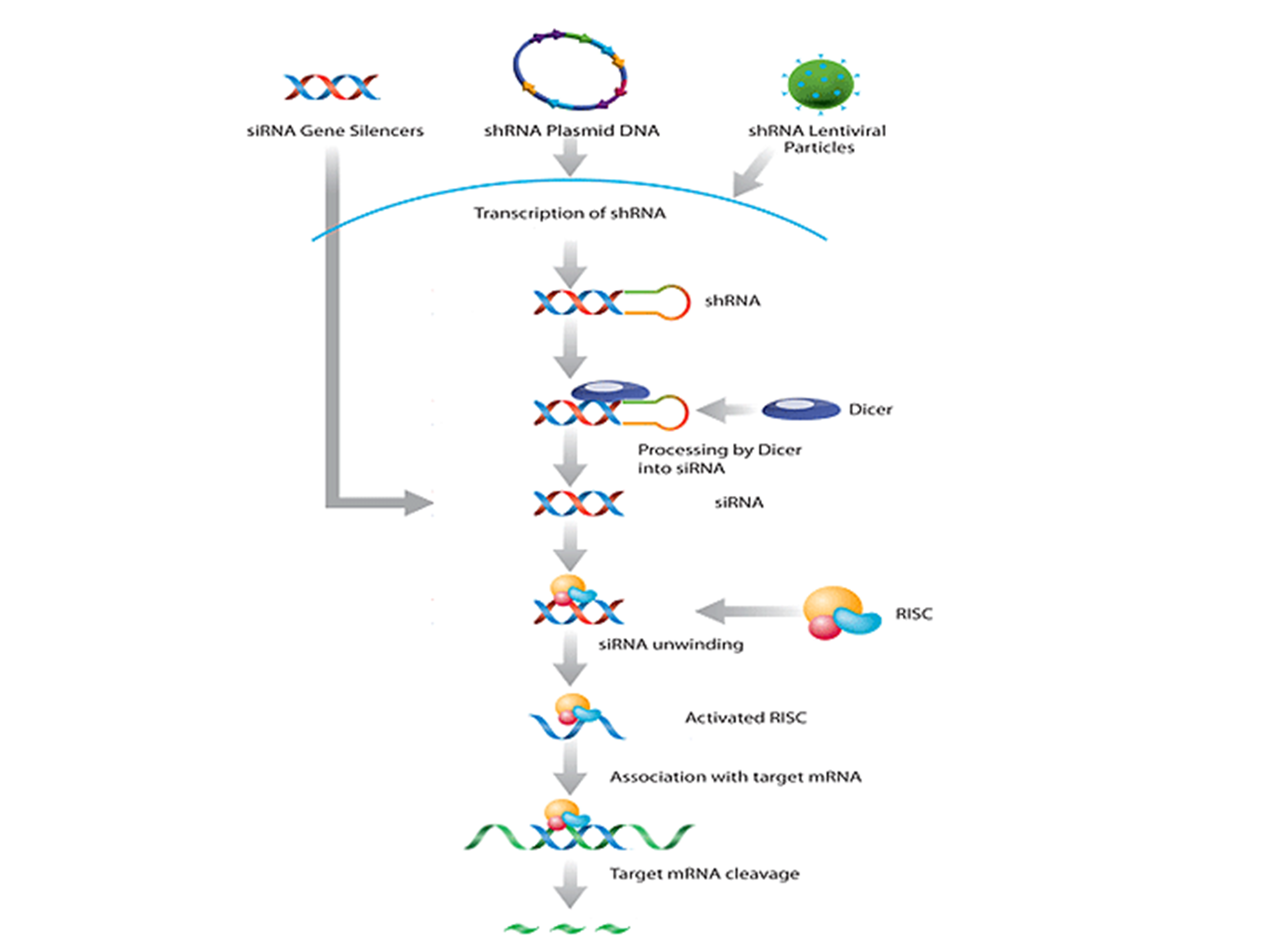

Supplement: Supplementary file 1 — Additional figure 1. [file CNS-29-2925-s003.tif]

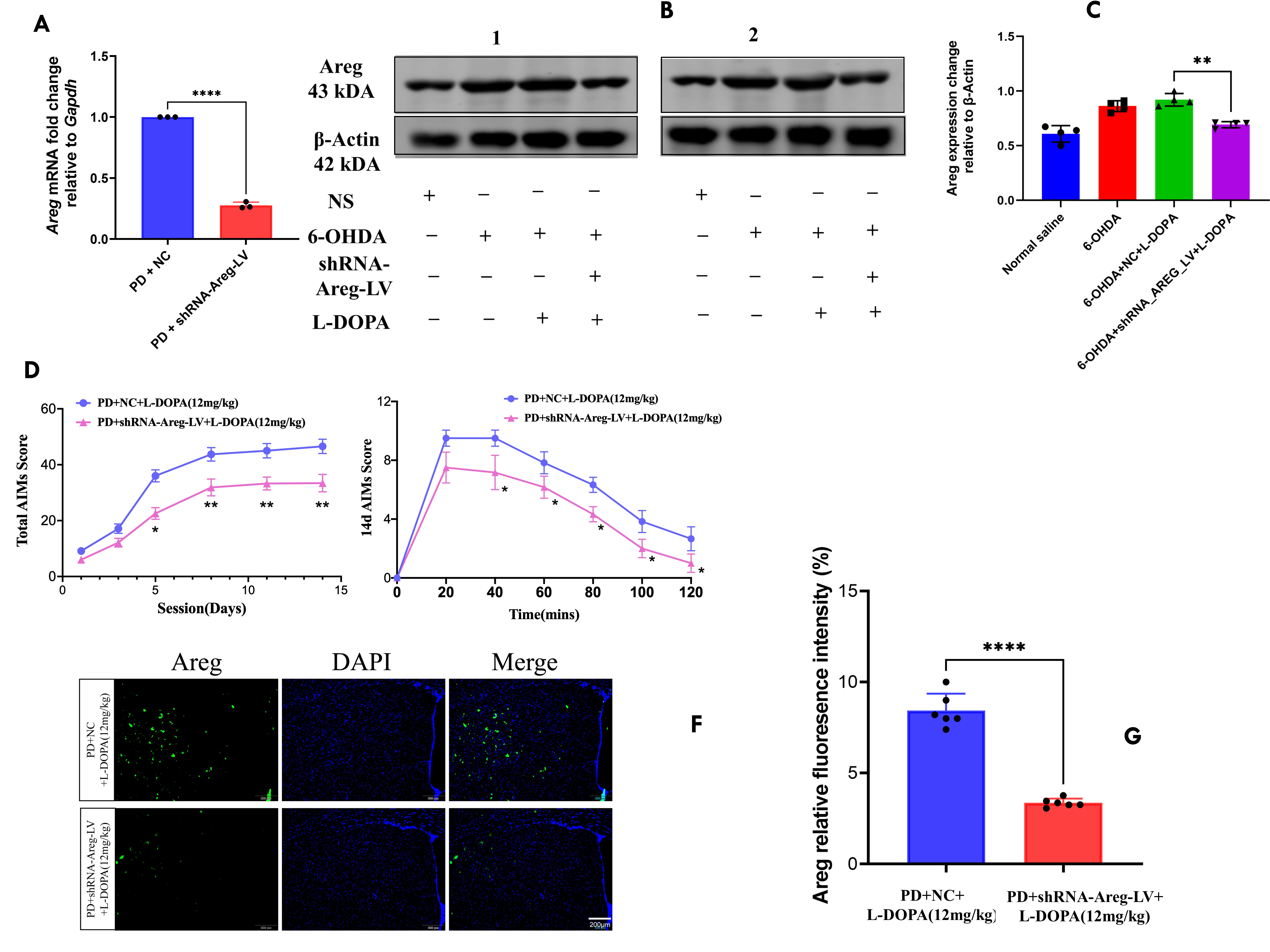

Supplement: Supplementary file 2 — Additional figure 2. [file CNS-29-2925-s004.tif]
